# Improving the Denominator of Safety Rankings for Bicyclist and Pedestrian Safety

**DOI:** 10.1007/s11524-026-01126-7

**Published:** 2026-08-21

**Authors:** Caitlyn Jeri Linehan, Trisalyn Nelson, Achituv Cohen

**Affiliations:** 1 Department of Geography, University of California, Santa Barbara, CA 93106 USA; 2 Department of Civil Engineering, Ariel University, Israel

**Keywords:** Active transportation safety, Injury prevention, Crash risk, Active transportation exposure, *K*-means, Volume

## Abstract

Active transportation increases physical activity and improves health, yet rising pedestrian and bicyclist deaths are a growing public health concern. In North America, regional agencies allocate transportation investments to support safe walking and bicycling. While comparing crash statistic across regions is standard, accurate safety assessment requires exposure data, or the volume of active transportation users, to determine crash rates. Calculating spatial variation in crash rates is difficult given the lack of spatially detailed, regionally extensive exposure data. Our goal is to develop a method to compare bicycling and pedestrian safety between cities, to inform decisions on where investments are most needed to reduce injury and death. We measured exposure as miles walked or biked in California’s 15 largest cities, using a validated model and data from the US Census, National Household Travel Survey, OpenStreetMap, and a statewide crash database. We performed linear regression with exposure and crashes, ranking cities by safety based on residuals. Higher positive residuals indicated less safe cities for active transportation. Including exposure shifted which cities had the highest safety risks for bicyclists and pedestrians. Safety differences were greatest in cities with high percentages of Hispanic residents, where crash-to-volume ratios showed unsafe conditions. Our method enables robust regional assessment of bike and pedestrian safety, allowing health departments and planners to identify where infrastructure investments can effectively prevent injuries and deaths, reduce health disparities, and create environments supporting active living. Our approach is scalable to major population centers nationwide, supporting evidence-based decisions that protect public health.

## Introduction

The public health burden of traffic-related pedestrian and bicyclists’ deaths is substantial and growing. Road traffic injuries represent a critical urban health challenge projected to become anywhere from the fifth to seventh leading cause of death globally by 2030 as urbanization and vehicle use continue to increase [1, 2]. In urban environments where pedestrians and bicyclists share roadways with motor vehicles, vulnerable road users face significantly elevated health risks, including increased rates of injury and mortality [3]. In the USA in 2021, there were 7388 pedestrians who faced traffic-related injuries or deaths which was a 12.5% increase from 6565 pedestrians in 2020 [4]. In 2020, 938 bicyclists died, which was a 9% increase from 859 bicyclists in 2019 [5]. The urban health burden of road traffic injuries from 1990 to 2019 was most severe in low- and middle-income countries, where bicycling injuries represented the leading urban transport-related health impact (34%) and pedestrian deaths were the primary cause of traffic-related mortality (37.4%) [6]. Between 2010 and 2020, Caltrans, the California Department of Transportation, put forward their own active transportation plan, in which they planned to utilize $1 billion in order to achieve health-protective goals that increase physical activity through active transportation while reducing injury and death [7]. Regional investments for programs and infrastructure that encourage safer active transit are often prioritized based on need, which is typically defined using unsafe walking or bicycling conditions.

A challenge in comparing regional safety for active transportation users, like bicyclists and pedestrians, is that crash data are often used without considering the volume of pedestrians and bicyclists. Volume, or exposure, refers to the at-risk denominator for a crash: the amount of walking or biking that could result in a collision, measured here as person-distance (miles walked or biked and analogous to person-time in epidemiology) [8, 9]. We use “exposure” distinct from its more common epidemiologic sense, where exposure refers to a risk factor such as cigarette smoking. Normalizing crashes by exposure yields an incidence rate, the number of crashes per unit of distance walked or biked [8, 9], which makes safety comparable across places with different levels of walking and biking. Without exposure data, it is hard to compare safety or risks across multiple places. For example, exposure data are needed to distinguish between a high number of crashes and high crash rates [9]. A high number of crashes could be due to higher ridership, while a high crash rate indicates a dangerous location and is comparable across multiple places. For instance, Ferster et al. (2021) mapped biking exposure to adjust for bike safety incident hotspots. They found that when incidents were normalized by exposure, unsafe bicycling areas shifted away from protected bike lanes in downtown to commercial streets outside the city core, where infrastructure was lacking.

Exposure data are limited, especially at the city or regional level, given counts of bicyclists and pedestrians tend to be point-based and limited in space and time. Modeled exposure has been helpful, but difficult to implement due to limited data. For example, Handy and Salon (2013) developed a method to estimate bicyclist and pedestrian activity at the census tract level of geography based on travel surveys, census data, and land use data. However, this model has not been used widely given challenges with data limitations. However, new open data are helping us overcome barriers, making it possible to map exposure regionally. One example of how lack of exposure data may be limiting decisions on active transportation investments is the California Office of Traffic Safety, which ranks cities based on crashes but lacks exposure data for characterizing crash rates. Inaccurate rankings may occur when cities have different levels of walking and bicycling. For example, two cities that each have 50,000 residents and 100 bicycle crashes each year may have a similar safety ranking. However, if one city has 1000 people who ride bicycles and the other has 10,000 people who ride bicycles, the rate of incidents is different.

Our goal is to develop a method to compare bicycling and pedestrian safety between cities, to inform decisions on where investments are most needed to reduce injury and death. To meet this goal, we (1) define a geographically sensitive exposure model by classifying census tracts based on their neighborhood type as rural, suburban, urban, and central city; (2) estimate active transportation exposure based on city-wide miles walked/biked using a validated model and diverse spatial data; (3) calculate comparable safety statistics, incorporating crashes and exposure, using a linear regression approach that emphasized residuals; and (4) quantify the characteristics of cities where safety measures showed increased risk when exposure was considered. We offer a method for regional evaluation of active transportation safety that normalizes safety statistics based on levels of walking and biking, enabling health officials and planners to implement evidence-based, health-protective interventions in large population centers across the USA.

## Methods

### Study Area

**Fig. 1:**
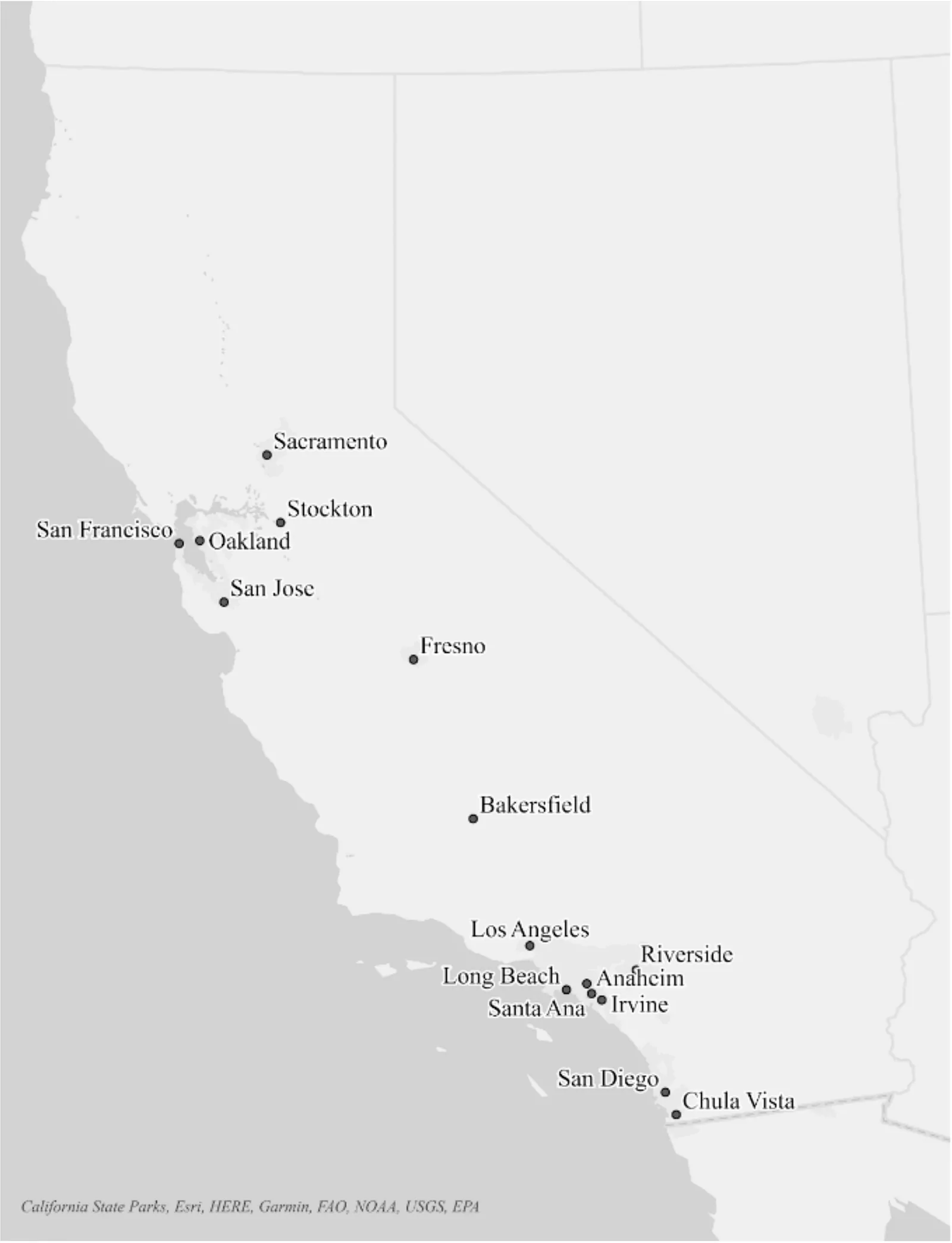
Study area of the 15 cities with the largest population in California

The study area is 15 cities within California with a population greater than 250,000: Anaheim, Bakersfield, Chula Vista, Fresno, Irvine, Long Beach, Los Angeles, Oakland, Riverside, Sacramento, San Diego, San Francisco, San Jose, Santa Ana, and Stockton (Fig. 1).

Table 1 shows some selected demographic and socioeconomic trends for our study area of the 15 largest cities in California from the 2020 American Community Survey. For these cities in California, the population ranged from 268,779 (Chula Vista) to 3,973,278 (Los Angeles). The median income ranged from $53,368 (Fresno) to $119,136 (San Francisco). The percentage of workers who commuted to work by walking ranged from 0.8% (Bakersfield) to 11.7% (San Francisco). The percentage of workers who commuted to work by biking ranged from 0.2% (Chula Vista) to 3.7% (San Francisco). The percentage of the population who identified as Hispanic ranged from 15.2% (San Francisco) to 76.0% (Santa Ana).

**Table 1:** Selected demographic and socioeconomic characteristics of California’s 15 most populous cities, 2016 to 2020

City	Total population	Median income ($)	Commuters who walked to work (%)	Commuters who biked to work (%)	Population who are Hispanic (%)
Anaheim	353,085	$76,723	1.6%	0.4%	53.3%
Bakersfield	379,879	$65,687	0.8%	0.3%	51.1%
Chula Vista	268,779	$86,132	1.6%	0.2%	60.3%
Fresno	526,147	$53,368	1.5%	0.4%	49.7%
Irvine	272,694	$108,318	4.3%	1.3%	10.7%
Long Beach	462,081	$66,410	2.8%	0.7%	43.2%
Los Angeles	3,973,278	$65,290	3.3%	0.8%	48.1%
Oakland	422,575	$80,143	3.6%	2.3%	27.0%
Riverside	327,569	$72,738	2.5%	0.6%	53.6%
Sacramento	503,482	$65,847	3.0%	1.8%	28.3%
San Diego	1,414,545	$83,454	2.9%	0.8%	30.1%
San Francisco	874,784	$119,136	11.7%	3.7%	15.2%
San Jose	1,029,409	$117,324	1.7%	0.6%	31.0%
Santa Ana	332,610	$72,406	2.1%	0.7%	76.0%
Stockton	311,103	$58,393	1.0%	0.3%	43.5%

All values from the American Community Survey 5-year estimates (2016 to 2020). Walk and bike commute percentages reflect the share of workers whose primary means of travel to work was walking or bicycling. Hispanic percentage reflects residents identifying as Hispanic or Latino of any race

### Classifying Census Tract by Neighborhood Type

Our first step was to categorize census tracts into neighborhood types (central city, urban, suburb, or rural**)** in order to develop geographically sensitive models of exposure. Data used included census data (American Community Survey 2016–2020), OpenStreetMap (OSM), and the Longitudinal Employer-Household Dynamics data (2020) from the Center for Economic Studies at the US Census Bureau (Table 2). We utilized similar factors for calculating neighborhood type to [11]; these built environment and socioeconomic variables can be used in discerning differences between neighborhood types. For census data, we utilized population density, median house value, percentage of old housing units, percentage of new housing units, percentage of single-family houses, percentage of commuters who walk or bike to work, and number of workers who lived in each census tract. From OSM, we utilized road density and the number of restaurants in each census tract. We utilized open-source crowdsourced data from OSM as this source has been shown to be very accurate when compared to authoritative datasets [12, 13].

**Table 2:** Input variables for the *k*-means classification of California census tracts into neighborhood types

Data name	Data piece	Data source	Year
popdens	Population density (people per square meter)	ACS^a^ and 2020 Census	2016–2020
medianvalue	Median value (dollars)	ACS^a^	2016–2020
pold	Percentage of old housing units	ACS^a^	2016–2020
pnew	Percentage of new housing units	ACS^a^	2016–2020
psfh	Percentage of 1-unit detached total housing units	ACS^a^	2016–2020
ptransit	Percentage of walk or bike commuters	ACS^a^	2016–2020
rdens	Road miles per square mile of census tract	OSM^b^	2023
jobaccess	Number of workers residing in each census tract	LEHD^c^	2020
restwwalk	Number of restaurants per census tract	OSM^b^	2023

All variables were standardized (mean 0, standard deviation 1) before clustering. Old housing units were built in 1949 or earlier; new housing units were built in 2000 or later

^a^*ACS*, American Community Survey 5-year estimates

^b^*OSM*, OpenStreetMap

^c^*LEHD*, Longitudinal Employer-Household Dynamics, US Census Bureau

Given that different geographies have different factors that govern walking and bicycling, we accounted for geographical context by classifying California census tracts into four different neighborhood types: rural, suburb, urban, and city center following the methodology from Handy and Salon (2013). We used the nine variables from a variety of datasets (Table 2). Census tracts from 2020 were used in order to match to other data sources where 2020 is the most recent available data. All of the analysis was done in R, and we scaled the data so that the mean of each column is zero and the standard deviation is one. For the *k*-means analysis, we used four clusters. For this work, we assigned the four clusters to represent the four different neighborhood types: rural, suburb, urban, and city center based on the variable averages. These clusters are distinctive from one another for the different built environment, demographic, and socioeconomic variables that we utilized in Table 2. Each census tract is assigned a different neighborhood type, and these types will later be used to determine representative values for miles walked/biked for different population/gender groups.

### Calculating City-Wide Active Transportation Exposure

Our second step was to utilize the neighborhood types we calculated, trip data from the National Household Travel Survey, and census data to estimate exposure (miles walked/biked) for the 15 cities in our study area (Table 3).

**Table 3:** Additional data sources used to estimate active transportation exposure (miles walked and biked) for the 15 study cities

Data name	Source	Year
Tract populations by gender and age group	ACS^a^	2016–2020
Demographics, trip descriptors, and trip location	NHTS^b^	2017

These sources supplement the variables in Table 2. Tract-level population counts were disaggregated by age group and gender to match the representative walking and biking values described in the text

^a^*ACS*, American Community Survey 5-year estimates

^b^*NHTS*, National Household Travel Survey

We used the National Household Travel Survey to calculate patterns in miles biked and walked in order to estimate exposure levels. The National Household Travel Survey contains a travel log in which respondents recorded the trips that they took over a 24-h period and by which mode that they traveled as well as trip length. Using this log, an additional table from the NHTS that contains demographics of travel respondents, and the spatial location of respondent’s residence, we extracted trips taken by walking or biking. We then geocoded the trips to assign the trip to the census tract that the respondent resided in and grouped trip data by the unique respondent identifier and summed the trip mile length to get the total miles walked or biked during a 24-h period per respondent. We excluded trips taken by a respondent who reported walking more than 9 miles in a day or biking more than 30 miles in a day [11]. To build a model of the amount of walking and bicycling, we determined representative values of the average miles walked/biked by respondents grouped by age, gender, and neighborhood type. First, we took the biking and walking trips that we prepared from the National Household Travel Survey as described above and grouped them by the following age groups: 5–14, 15–29, 30–49, 50–64, 65–74, and 75 and over as well as by gender and neighborhood type. The grouping resulted in 48 representative values for average miles walked by gender, age group, and neighborhood type and 48 representative values for average miles biked.

In order to calculate an exposure estimate of total miles walked or biked for the 15 cities in our study area, we used the representative values and the population totals per census tract. For each census tract, we gathered the population numbers of the different age and gender groups as explained above. Next, using the neighborhood type assigned to each census tract, we attached the representative values of the 12 age and gender categories. For each of the age groups for males and females, we calculated the total miles walked/biked per that age and gender group. We then summed the total miles walked/biked for the different age and gender groups to calculate the active transportation exposure values: the total miles walked/biked per census tract. We then summed the total miles walked/biked per city using all the individual census tract exposure values within the city boundaries.

### Adjusting Safety Statistics

For the third step, we used crash data in our regression analysis, as well as the total miles walked/biked aggregated from the previous step, to demonstrate how to determine a comparative analysis of city-wide bike and pedestrian safety statistics. We downloaded the total amount of pedestrian and bike crashes, including both severe and non-severe crashes based on injury level, from the Transportation Injury Mapping System (TIMS) from SafeTREC at UC Berkeley in 2020.

We demonstrate how the use of active transportation exposure changes representations of safety statistics using a case study of the California Office of Traffic Safety (OTS). With the aim of adjusting rankings, we performed linear regression analyses and utilized residuals in normalizing safety statistics. We calculated the sum of total miles walked, sum of all pedestrian crashes, sum of total miles biked, and sum of all bike crashes. Next, we performed two linear regression analyses: one for pedestrian exposure and crashes and one for bicyclist exposure and crashes. The predictors are the total miles walked/biked, respectively, and the outcomes are total pedestrian and bike crashes. We chose a linear regression model to see whether an increase in active transportation exposure is associated with an increase in crashes. Crash counts are often modeled with Poisson regression, using an offset for exposure to estimate a rate [14, 15]. Furthermore, we chose linear regression because we rank cities by how far their crash counts depart from what their exposure predicts, and the linear residuals give us this deviation directly, in the original units of crashes. Thus, we then used the residuals from the linear regression model to create a ranking system in which negative residuals or lower residuals indicated a safer city for biking/walking and were assigned higher values. Conversely, positive and higher residuals indicated a less safe city for biking and walking and were assigned lower ranking values. We used exposure (total miles walked or biked) as the only predictor in these models. We did not include built environment or socioeconomic variables because they would explain away part of the residual, and we use the residual to rank relative safety. The residual shows how many more or fewer crashes a city has than its exposure alone would predict. Our goal is to flag cities with more crashes than expected given their levels of walking and biking. Our choice also keeps the method scalable, so that any city or region can apply it using crash data and exposure estimates, without relying on detailed built environment data that are often inconsistent or missing across places.

### Contextual Information for Equity Implications

For the fourth step, we assessed the relationship between the safety statistics and demographic variables among the 15 cities in order to explore equity considerations of access to safe active transportation. We gathered the percentage of Hispanic residents for each of the 15 larger cities of California using the American Community Survey (2016–2020). We then calculated the median percentage of Hispanic residents among the 15 cities and bisected the cities depending on if they are greater than the median or less than or equal to the median. We integrated this demographic factor as recent research has shown that not all population groups have equal safety experiences when biking or walking.

## Results

### Classifying Census Tracts by Neighborhood Type

In Table 4, we demonstrate the built environment, demographic, and socioeconomic variables that differentiate the neighborhood types (rural, suburb, urban, and central city) for all of the census tracts within California that we will later use in differentiating exposure values. We present the standardized means of the variables in each neighborhood cluster (rural, suburb, urban, and central city) and how many census tracts within California are within each cluster (Table 4). The four neighborhood types are distinct across the classification variables (Table 4). For example, central city tracts score well above the state average in population density, restaurant count, road density, and walk or bike commuting.

**Table 4:** Standardized mean values of classification variables for each neighborhood type, California census tracts

Variables	Rural^a^	Suburban^a^	Central city^a^	Urban^a^
Restaurants in tract	−0.19	−0.19	2.73	−0.05
Percent walk/bike commuters	−0.40	−0.23	2.65	0.09
Percent single family detached houses	0.66	0.47	−1.47	−0.71
Percent new housing	2.56	−0.23	0.21	−0.37
Percent old housing	−0.69	−0.37	0.84	0.65
Median house value	−0.27	−0.16	0.97	0.19
Population density	−0.57	−0.44	1.21	0.67
Jobs	0.05	−0.09	0.72	0.03
Road density	−0.56	0.47	1.56	0.67

Values are standardized across all California census tracts (mean 0, standard deviation 1), so each entry shows how far that neighborhood type departs from the statewide average in standard deviation units. Positive values are above the state average and negative values below. For example, central city tracts are 1.21 standard deviations above the mean in population density, and rural tracts are 0.57 below

^a^Number of California census tracts in each neighborhood type: rural, 818; suburban, 4758; central city, 480; urban, 3073

### Calculating City-Wide Active Transportation Exposure

We present representative active transportation exposure values from the different age/gender/neighborhood types that we gathered from the National Household Travel Survey (Tables 5 and 6). The greatest amount of miles walked are for males and females between the ages 50–64 and males aged 65–74. The least amount of miles walked are for males and females between the ages 5–14 and for females aged 75 and over. The greatest amount of miles biked are for males between the ages of 30–49, 50–64, and 65–75 and for women between the ages of 50 and 64. The least amount of miles biked are for males and females between the ages of 5–14 and greater than 75.

**Table 5:** Mean daily miles walked per person by age group, gender, and neighborhood type, 2017 National Household Travel Survey

Walking group	Central city	Urban	Suburban	Rural
5–14 (male)	0.67	1.01	1.06	0.96
5–14 (female)	0.81	0.97	0.99	0.75
15–29 (male)	1.24	1.39	1.58	1.48
15–29 (female)	1.45	1.24	1.43	1.62
30–49 (male)	1.6	1.24	1.28	1.55
30–49 (female)	1.56	1.37	1.3	1.25
50–64 (male)	1.52	1.54	1.55	1.85
50–64 (female)	1.51	1.47	1.47	1.51
65–74 (male)	1.72	1.75	1.46	1.6
65–74 (female)	1.48	1.55	1.28	1.38
75 and over (male)	1.65	1.23	1.18	1.64
75 and over (female)	0.93	0.96	1.11	0.83

Values are representative mean daily miles walked per person, derived from NHTS 2017 trip logs and grouped by age, gender, and neighborhood type. These values were combined with tract-level population counts to estimate total miles walked per city. Trips exceeding 9 miles walked in a day were excluded. NHTS, National Household Travel Survey

**Table 6:** Mean daily miles biked per person by age group, gender, and neighborhood type, 2017 National Household Travel Survey

Biking group	Central city	Urban	Suburban	Rural
5–14 (male)	1.63	2.46	1.90	1.95
5–14 (female)	1.62	2.54	1.42	1.90
15–29 (male)	4.95	4.18	5.67	5.60
15–29 (female)	5.45	4.44	3.85	3.96
30–49 (male)	4.17	4.53	6.56	9.09
30–49 (female)	3.66	4.84	4.45	2.89
50–64 (male)	6.07	7.11	8.36	6.20
50–64 (female)	4.08	5.27	7.67	5.63
65–74 (male)	5.33	7.96	6.86	9.35
65–74 (female)	1.87	4.99	4.82	5.03
75 and over (male)	2.76	3.21	5.77	5.43
75 and over (female)	0.15	0.70	4.64	0.00

Values are representative mean daily miles biked per person, derived from NHTS 2017 trip logs and grouped by age, gender, and neighborhood type. These values were combined with tract-level population counts to estimate total miles biked per city. Trips exceeding 30 miles biked per city. Trips exceeding 30 miles biked in a day were excluded. NHTS, National Household Travel Survey

### Adjusting Safety Statistics

**Fig. 2:**
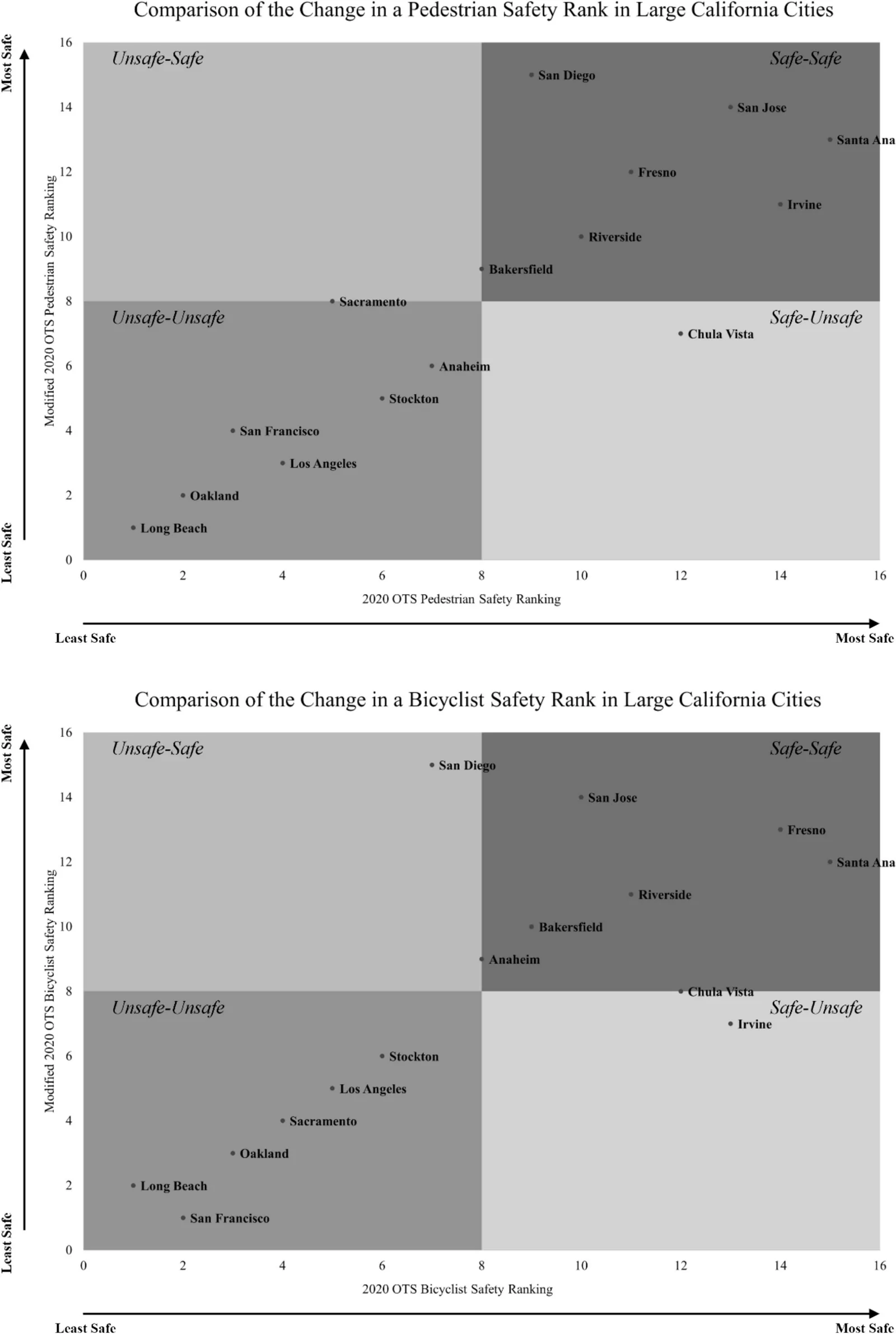
Scatter plot that shows how city ranks have changed in the current and modified pedestrian (top) and bicyclist (bottom) rankings

For our linear regression models for both pedestrians and bicyclists, we had *R*^2^ values of 0.95 and 0.94, respectively. We show the results of the city-wide comparative analysis of pedestrian and bike safety statistic changes for large cities in California with the inclusion of active transportation exposure (Fig. 2). Chula Vista and Irvine had the largest decrease in both the pedestrian and biking safety statistic rankings. Sacramento and San Diego had the largest increase in the pedestrian safety statistic ranking, while San Diego and San Jose had the largest increase in the biking safety statistic ranking. In order to demonstrate how city rankings have changed in Fig. 2, we present quadrant plots in which the *x*-axis shows the current pedestrian/bicyclist rank and the *y*-axis shows the modified (new) pedestrian/bicyclist rank. For both the current and modified rankings, cities are ordered from 1 to 15, where 1 denotes the lowest safety rank and 15 the highest. In these plots, the low-high quadrant shows cities that have a current ranking below the mean (thus less safe) and with the modified ranking above the mean (thus becoming safer). The low-low quadrant shows cities that have a current ranking below the mean (thus less safe) and with a modified ranking below the mean (thus remaining less safe). The high-high quadrant shows cities that have a current ranking above the mean (thus safer) and with a modified ranking above the mean (thus remaining safer). The high-low quadrant shows cities that have a current ranking above the mean (thus safer) and with a modified ranking below the mean (thus becoming less safe). The pedestrian metric changes show that Chula Vista is a city that was previously safer but with the modified ranking became more unsafe. With the inclusion of active transportation exposure, San Diego is a city that was already considered rather safe but is now considered even more safe for pedestrians. The bicyclist metric changes show that San Diego is a city that was previously unsafe but is now considered safer for bicyclists. Conversely, Irvine was considered a safer city for bicyclists with the previous ranking but with the current ranking is considered more unsafe.

### Contextual Information for Equity Implications

**Fig. 3:**
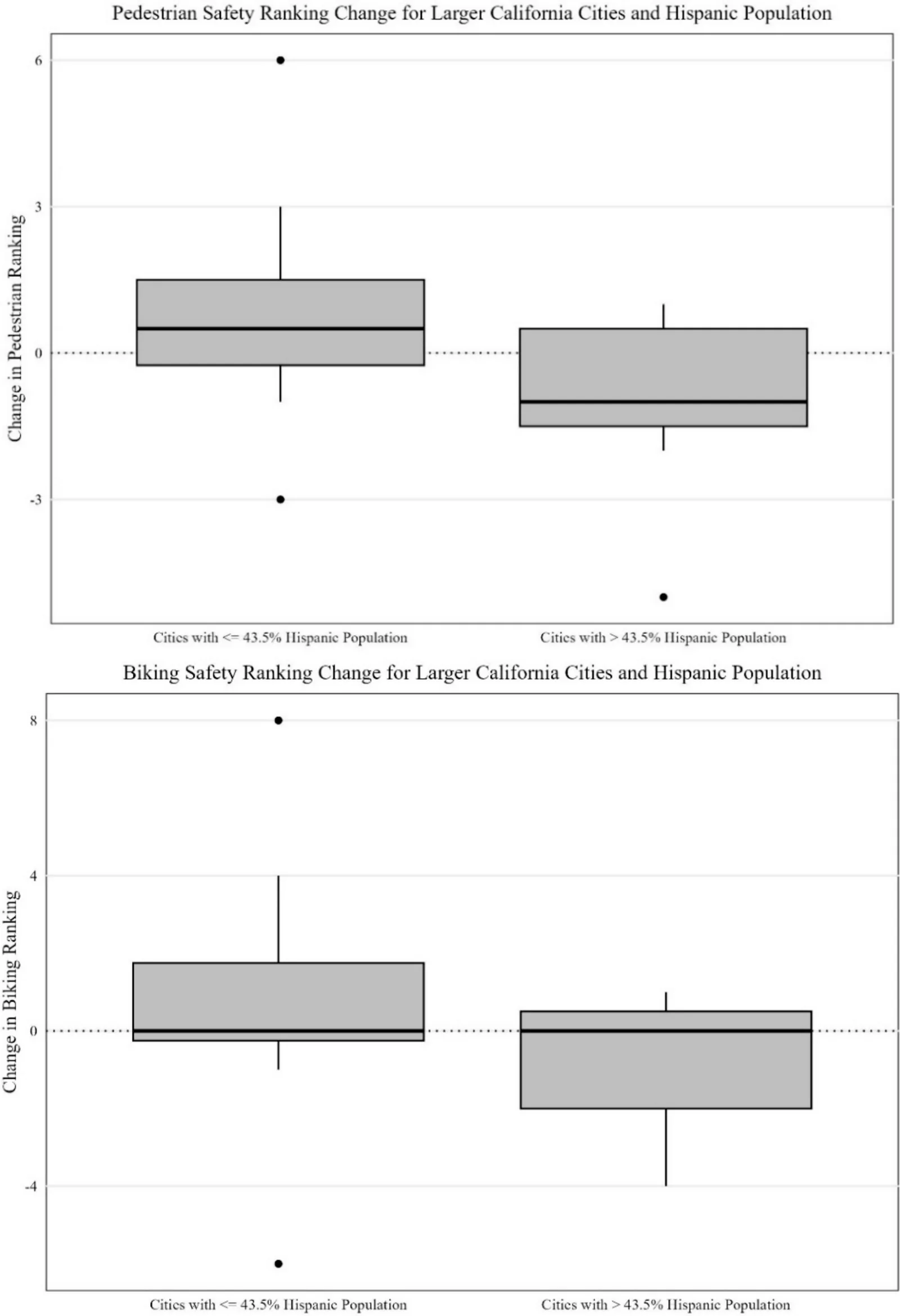
Box plots showing the change in pedestrian (top) and bicyclist (bottom) safety rankings bisected by their Hispanic population percentages. Cities above the median percentage of Hispanic residents are compared to those below the median, illustrating differences in safety rank changes across these two groups

In the plots in Fig. 3, we demonstrate the change in the pedestrian and bicyclist safety ranking for two groups: cities with less than or equal to 43.5% of Hispanic residents and cities with greater than this percentage (the median percentage of Hispanic residents among the 15 cities). Cities with a higher than median percentage of Hispanic residents often experienced a decrease in the safety rankings, thus becoming more unsafe.

## Discussion

Our work highlights the importance of incorporating exposure when evaluating health risks of unsafe walking and bicycling across a region. Our findings demonstrate that including active transportation exposure can significantly alter how a region’s injury burden and health risks are measured and perceived. For example, while Chula Vista was previously considered safer for walking compared to other major cities in California, when accounting for active transportation exposure, it is comparatively less safe for pedestrians. Given the estimated number of pedestrians in Chula Vista, we would expect a lower incidence of crashes; however, the residuals from our linear regression models indicate that actual crash numbers are higher than anticipated, suggesting elevated health risks for walkers. The revised safety rankings are essential for informing health-protective policies and targeted injury prevention interventions. Previous biking research has shown that including active transportation exposure in safety analyses can shift the location of crash hotspots, such as moving from bike lanes in downtown areas to commercial streets in the city’s peripheral [10]. By incorporating active transportation exposure into health and safety assessments, health and transportation officials can better understand not only where people are walking or biking but also identify areas where crashes and injuries are disproportionately occurring relative to the levels of exposure which enables targeted interventions where they are needed to save lives and reduce injury.

The inclusion of active transportation exposure in safety statistics is crucial for health equity, as it helps identify which cities and populations face the greatest injury risks while walking and biking. In low-income urban neighborhoods, particularly those with large Black and Brown populations, there is often a lack of infrastructure that supports safe active transportation, creating health disparities. Research indicates that certain populations, such as Black and Hispanic pedestrians under the age of 16, Black and Native American pedestrians during late hours, and Asians over the age of 65, are often disproportionately involved in crashes and face a large proportion of injury burdens [16]. Additionally, studies have shown that Black and Hispanic pedestrians and bicyclists are more likely to be involved in crashes [17]. Beyond direct injury risks from crashes, urban traffic–generated PM_2.5_ pollution poses serious cardiovascular health threats to city residents by triggering oxidative stress, inflammation, and vascular damage that may lead to hypertension, heart disease, and strokes [18]. Rush hour exposure peaks create concentrated health risks in densely populated urban areas, particularly affecting underserved populations in low- and middle-income cities [18]. As shown in Fig. 3, cities with higher percentages of Hispanic residents are often perceived as having greater injury risks for active transportation users when active transportation exposure is factored into safety statistics. Our results underscore the importance of including exposure for advancing health equity, as it enables an investigation into access to safe active transportation infrastructure. Without considering exposure, public health and city officials may focus injury prevention resources on areas with high crash rates where active transportation infrastructure already exists, rather than addressing areas where unsafe conditions create the greatest health threats. Speed cameras and traffic calming interventions are highly cost-effective urban health strategies that substantially reduce road-related deaths and injuries in cities [19]. Including exposure allows officials to identify areas where people are unable to gain the health benefits of active transportation safely due to infrastructure gaps. Researchers have demonstrated that health-promoting active transportation infrastructure is often concentrated in more advantaged communities, leaving underserved populations without safe options for physical activity [20]. Further studies have shown that communities with high proportions of Black, Hispanic, and Asian residents, low-wage employees, those with lower educational attainment, and older individuals often lack access to bike infrastructure [21]. By incorporating active transportation exposure into safety statistics, we can produce more accurate health risk assessments that highlight inequities in both injury burden and access to infrastructure that supports healthy, active living. Australia’s public health approach to road safety included mandatory seatbelt laws, random breath testing, and strategic speed enforcement which successfully reduced traffic-related injuries and deaths in cities through targeted behavioral interventions [2], demonstrating that comprehensive, evidence-based policies can effectively address transportation-related health disparities.

Future research can benefit from emerging pedestrian and bicycling datasets, as well as new methodologies for estimating active transportation exposure to improve public health risk assessments [9, 11, 22, 23]. Location services within apps, when curated by companies like Streetlight, can generate valuable metrics for pedestrians and bicyclists, including origin–destination demand, trip attributes, and aggregated traveler data [24]. While these location-based datasets offer innovative opportunities for active transportation mobility research, they also present several challenges. The costs of acquiring such data can be prohibitive, and privacy concerns mean that individual traveler attributes are often not available. Additionally, the data may not specify the mode of transport, making it difficult to differentiate between modes. For example, a slow biking trip might be misclassified as a walking trip [25], which hinders accurate active transportation exposure calculations. Another promising data source comes from fitness-tracking apps, which collect physical activity data, such as walking and biking, via users’ mobile devices. While these apps provide valuable information, they suffer from sample bias, as they tend to attract recreational users rather than offering a representative sample of the broader population [25]. Despite these challenges, these emerging datasets present a significant opportunity for future research into active transportation exposure and safety statistics. However, further work is needed to correct for biases within these data sources.

## Limitations

The research presented here is conducted on a large statewide scale, and as with any such work, certain assumptions and choices had to be made in the modeling process, which we discuss below. In generating the representative values shown in Tables 5 and 6, we assigned the miles walked and biked to the tract where the respondent resided. This assumption is based on the premise that individuals primarily walk and bike near their homes. As a result, our model reflects the activity patterns of residents, rather than capturing those of visitors passing through the tract. Additionally, when creating representative values for miles walked and biked, we consider variables such as age, gender, and neighborhood type. Due to the smaller sample size, however, we are unable to disaggregate data by racial or ethnic groups, meaning we do not account for potential differences in active transportation levels among these groups. Given these limitations, it is important to note that city and state officials often require state, region, or city-wide data to estimate safety issues. This paper demonstrates how such large-scale safety estimates can be made, but it also highlights the limitations that arise from the assumptions and decisions necessary for this scale of analysis. Future research should aim to estimate active transportation exposure at the segment level and include data on racial and ethnic differences in walking and biking rates to improve the precision and inclusivity of these safety and health risk assessments.

Furthermore, our linear regression models show high *R*^2^ values, that indicates for larger cities in California, exposure strongly predicts the number of crashes. However, these findings should not be taken to suggest that built environment infrastructure interventions are inconsequential. Rather, the *R*^2^ values suggest that exposure is a driver of absolute crash numbers. Furthermore, the value of our study lies in the residuals: cities with large positive residuals experience more crashes than their exposure values would predict, signaling unsafe conditions that interventions could address. Cities with crashes that were near predicted levels, or had smaller residuals, signal cities where safer conditions are present. We also acknowledge that city-level aggregation likely inflates our model fit compared to more granular spatial analyses, and it may also mask within-city variation. Our city-level approach is suitable for regional prioritization of safety investments and identification of broad health disparities and should complement corridor-level safety analyses.

## Conclusion

We present an approach that integrates diverse spatial data to calculate pedestrian and bicyclist exposure regionally, normalizing crash and injury risk across 15 cities in California to emphasize the importance of assessing safety and planning injury prevention interventions. Our adjustments show how regions’ safety statistics change when exposure is included, providing a more accurate picture of health risks. We also found that areas with more Hispanic residents face disproportionately higher injury risks in the adjusted statistic, revealing important health disparities. This work shows how exposure can promote health equity in access to active transportation by helping public health and transportation officials identify areas with high crash rates and distinguish between typical rates and those that are unusually high, which indicates a need for health-protective infrastructure investments. By incorporating active transportation exposure into the normalization of safety statistics, we are able to uncover health inequities within regions that might otherwise remain hidden. Our method scales health risk analysis, enabling regions to accurately compare active transportation injury burden between cities and thus allocate prevention resources where they can reduce injury and death. While similar assessments are common at the street level, inter-city comparisons are essential for guiding regional public health investments and creating conditions that allow all residents to safely gain the health benefits of walking and biking.

## Data Availability

The data in our study are derived from a combination of publicly available and restricted- access sources. Pedestrian and bicyclist crash counts were obtained from the Transportation Injury Mapping System (TIMS), Safe Transportation Research and Education Center (SafeTREC), University of California, Berkeley. Population and demographic data were obtained from the U.S. Census Bureau, including the American Community Survey (2016-2020). Street network data were obtained from OpenStreetMap. Active travel behavior data were drawn from the National Household Travel Survey, administered by the Federal Highway Administration, which are not publically available due to access restrictions. These data are available from the Federal Highway Administration subject to an application and data agreement.
